# FAMILY STRENGTH AND LIFE SATISFACTION IN OLDER ADULTS WITH VISUAL IMPAIRMENTS: A STRUCTURAL EQUATION MODELING

**DOI:** 10.1093/geroni/igae098.4191

**Published:** 2024-12-31

**Authors:** Eunjin Yang, Seohyeon Kim

**Affiliations:** Gachon University, Incheon, Republic of Korea; Seoul National University, Seoul, Republic of Korea

## Abstract

Although family strength has been recognized as one of the crucial factors in the well-being and life satisfaction of people with disabilities, studies regarding its specific role in the life satisfaction of older adults with visual impairment are still limited. Therefore, this study aimed to explore how family strength affects life satisfaction, examining the mediating roles of self-esteem and disability acceptance in this relationship. Data were obtained from the 4th Disability and Life Dynamics Panel in South Korea, analyzing 386 participants aged 50 and above. Structural equation modeling was employed to analyze the direct and indirect effects among family strength, self-esteem, disability acceptance, and life satisfaction. The overall model fit indices met the recommended criteria, indicating the hypothesized model appropriately represented the relationships between family strength and life satisfaction among older adults with visual impairments (Normed χ2=1.876, TLI=.985, CFI=.993, RMSEA=.048, SRMR=.017). The results revealed that family strength enhances life satisfaction, both directly and indirectly through promoting self-esteem and disability acceptance. These findings suggest that an integrated approach focusing on strengthening family support, as well as enhancing self-esteem and disability acceptance, is recommended to improve life satisfaction for older adults with visual impairment. This study contributes to a better understanding of mechanisms underlying life satisfaction in older adults with visual impairments. In addition, it provides valuable insights for developing targeted interventions to enhance family strengths and to create supportive strategies for this vulnerable population.

